# Impact of patient phenotype on the relationship between accelerometer-derived physical activity and cardiovascular events in atrial fibrillation

**DOI:** 10.1016/j.ajpc.2025.101362

**Published:** 2025-12-05

**Authors:** Maxime Boidin, Benjamin JR Buckley, Gregory YH Lip, Dick HJ Thijssen

**Affiliations:** a Department of Sport and Exercise Sciences, Institute of Sport, Manchester Metropolitan University, Manchester, United Kingdom; b Cardiovascular Health Sciences Research Group, Research Institute for Sport and Exercise Sciences, Liverpool John Moores University, Liverpool, United Kingdom; c Liverpool Centre for Cardiovascular Science at University of Liverpool, Liverpool John Moores University and Liverpool Heart & Chest Hospital, Liverpool, United Kingdom; d Danish Center for Health Services Research, Department of Clinical Medicine, Aalborg University, Aalborg, Denmark; e Department of Medical BioSciences, Cardiovascular Physiology, Radboud university medical centre, Nijmegen, the Netherlands

**Keywords:** Atrial fibrillation, Physical activity, Major adverse cardiovascular events, Af-related complications, Accelerometer, Phenotype-specific management

## Abstract

**Aims:**

Atrial fibrillation (AF) is a heterogeneous condition with varying comorbidity profiles, yet current physical activity guidelines do not differentiate between AF phenotypes. We examined the association between objectively measured moderate-to-vigorous physical activity (MVPA) and major adverse cardiovascular events (MACE) across different AF phenotypes.

**Methods:**

In a prospective cohort of 4858 adults with AF (mean age 63, 37 % female) from the UK Biobank, wrist-worn accelerometry quantified MVPA. Hierarchical clustering classified individuals into 'high' and 'low' risk clusters based on comorbidities. Activity patterns were also described by rhythm control treatment status. Cox-proportional hazards models assessed the association between MVPA and MACE across clusters, while Poisson regression identified notable MVPA thresholds.

**Results:**

The 'high-risk' cluster (*n* = 2583) experienced more MACE (HR: 3.81, 95 %CI: 3.19–4.55, *p* < 0.001) than the 'low-risk' cluster (*n* = 2275). Greater MVPA was associated with lower MACE incidence in both clusters. In the 'low risk' cluster, those with median MVPA of 187 min/week had lower MACE incidence (HR 0.39, 95 %CI 0.22–0.70, *p* = 0.001) than the reference group (median 56 min/week). In the 'high risk' cluster, those with median MVPA of 167 min/week had lower MACE incidence (HR 0.57, 95 %CI 0.44–0.74, *p* < 0.001) than their reference group (median 42 min/week). Poisson models identified 35 and 103 min/week as notable thresholds (IRRs: 0.35 and 0.31, respectively; both *p* < 0.001). Among patients undergoing rhythm control (*n* = 1354), higher MVPA was associated with lower MACE incidence (HR 0.42, 95 %CI 0.26–0.66, *p* < 0.001).

**Conclusion:**

In this AF cohort, higher MVPA was associated with lower MACE incidence across different risk phenotypes and treatment statuses.

## 1 Introduction

Atrial fibrillation (AF) is the most common clinically significant arrhythmia, affecting >33 million individuals worldwide [1]. The presence of AF leads to cardiac remodelling, and is strongly related to stroke [2]. heart failure (HF) and death [3]. Higher physical activity levels are associated with lower risks of incident AF (i.e., primary prevention) [4]. For example, moderate-vigorous intensity physical activity (MVPA) of at least 150 min/week was associated with 16 % lower risk in incident AF [5]. Similarly, previous work also revealed a protective role of regular MVPA in the secondary prevention of AF-related complications (e.g., stroke, HF, death, myocardial infarction [MI]) [6]. Moreover, a 2024 Cochrane review further supported the potential health benefits, as exercise-based cardiac rehabilitation resulted in significant improvements in AF burden, recurrence, and quality of life, although no effects were observed for all-cause mortality and serious adverse events [7]. Additionally, a UK Biobank study found that exceeding current MVPA guidelines (>150 min/week) was associated with lower risks of all-cause mortality and HF in individuals with AF [8].

However, a key limitation of previous research is the assumption that AF is a homogenous condition. In reality, AF encompasses a highly heterogeneous population, with individuals presenting varying degrees of cardiovascular risk, comorbidities, and disease progression. This heterogeneity is critical in clinical decision-making, as patients with different AF phenotypes may respond differently to interventions [9], including exercise. For example, previous work suggested the presence of cardiovascular risk and/or disease alters the relationship between MVPA and the risk for developing MACE [10,11]. Other works suggested that the risk of MACE might depend on sex and the presence of AF [12,13]. This raises an important clinical question: do the benefits of physical activity differ across AF phenotypes? Therefore, the primary aim of this study was to describe the association between MVPA and major adverse cardiovascular events (MACE) across different cardiovascular risk profiles for individuals with AF. For this purpose, we used phenotype classification based on a clustering process to identify a 'high risk' and 'low risk' phenotype within the UK Biobank dataset. An important advantage of the UK Biobank is that MVPA was measured using objective accelerometry, which provides more accurate data than the frequently used self-reported questionnaire data [14]. This allows for the characterization of the relationship between device-measured MVPA and major adverse cardiovascular events (MACE: cardiac arrest, strokes, HF, chronic heart disease, MI) between clusters, and the identification of notable MVPA thresholds. Another relevant AF phenotype relates to those undergoing rhythm control procedure. Therefore, as a secondary aim, we described the MVPA patterns and MACE incidence in individuals with AF who underwent a rhythm control procedure (i.e., cardioversion or catheter ablation). We sought to characterize the relationship between different levels of MVPA and MACE incidence in individuals with AF across different risk phenotypes and treatment approaches.

## 2 Methods

This study is reported as per the Strengthening the Reporting of Observational Studies in Epidemiology (STROBE) guideline (Supplemental Material). Further information are detailed in Supplemental Material.

### 2.1 Study participants

The UK Biobank is a prospective cohort that enrolled 502,369 individuals between 2006 and 2010 [15]. A total of 9.2 million individuals aged 40–69 years living within 25 miles radius of 22 assessment centres in the UK were invited, and 5.4 % participated in the baseline assessment. For the primary aim, all individuals with AF with objective assessment of physical activity were included. For the secondary aim, only individuals with AF who underwent a rhythm control procedure (i.e., catheter ablation or cardioversion) with device-measured physical activity were included. This subgroup reflects patients exposed to rhythm control procedures, but not necessarily those who achieved or maintained long-term rhythm control. Individuals diagnosed with AF or flutter (as previously undertaken [5]) were included. Physical activity data were collected over 7-days and clinical outcomes through national health-related dataset linkage. Written informed consent was obtained from all participants. The UK Biobank (ID 92,337) was approved by the UK Biobank Research Ethical Committee (reference number 11/NW/0382).

### 2.2 Clustering process

A hierarchical clustering process was undertaken using the Ward minimum variance method. The squared Euclidean distance was used as a measure of distance or dissimilarity, since only dichotomous variables were selected. The aim of this analysis was to identify the optimal number of clusters that were homogenous and indicative of a clinically relevant phenotypic subgroup of individuals with AF without a priori knowledge of the outcomes (see Supplemental Material for more details). Thirty one variables were used for the clustering process: age, sex, body mass index (BMI), ethnicity, alcohol and smoking habits, estimated glomerular filtration rate (eGFR), liver function, cardioversion history, catheter ablation of AF, LBBB, left ventricular hypertrophy, history of chronic obstructive pulmonary disease (COPD), chronic kidney disease (CKD), HF, sleep apnoea, malignancy, peripheral artery disease (PAD), dementia, strokes, heart disease, type 2 diabetes (T2D), hypertension, percutaneous coronary intervention (PCI), coronary artery bypass graft surgery (CABG), pacemaker, gastrointestinal (GI) bleeding, renal replacement therapy (RRT)/kidney transplantation, hypothyroidism, hyperthyroidism, and anaemia. Continuous variables were dichotomized according to usual clinical practice, to make all variables categorical, as age <60 and ≥60 years, BMI <25 kg/m^2^ (normal BMI) and ≥25 kg/m^2^ (overweight/obese), eGFR <90 (abnormal) and ≥90 (normal), anaemia characterised by haematocrit (threshold of 37 and 40 % for female and male, respectively), and liver function characterised by gamma-glutamyl transferase (≤40 U/L as normal). Missing values (*n* = 1344) were imputed, and include BMI, alcohol and smoking habits, cardioversion ad catheter ablation history, history of PCI, CABG, pacemaker, RTT/kidney transplantation, anemi, eGFR, dyslipidaemia, and liver function. A dendogram was used to display the clusters according to the Euclidean distance. After the display of the dendrogram, an appropriate number of clusters was determined by the decision whether the final number of clusters represented a homogenous group with relatively similar characters [9]. The clustering algorithm starts with each individual as an individual cluster and subsequently combines the most similar clusters together until only one cluster remains. This process is shown in a dendrogram, where the height of the lines indicates dissimilarity between clusters.

### 2.3 Clinical characteristics

Age, sex, and anthropometric measures were attained during initial study visits. Smoking status, alcohol intake, and use of medication were evaluated by self-reported touch-screen questionnaires where help was available. Clinical comorbidities were recorded across hospital inpatient records.

### 2.4 Clinical outcomes

The primary outcome was the first occurrence of MACE, which included cardiac arrest, strokes, HF, MI, and ischemic heart disease that occurred after both the diagnosis of AF and the accelerometry assessment, and up to a maximum of 6-years follow-up from index (defined as the later of AF diagnosis (primary analysis) or accelerometry (Figure S1), or AF procedure (subgroup analysis). We excluded all outcomes that occurred on the day of diagnosis of AF. Refer to Supplemental Table S3 for clinical codes.

### 2.5 Accelerometer-derived physical activity

Physical activity data extraction, calibration and cleaning has been detailed previously [16]. From May 2013 to December 2015, 28,476 individuals with AF were mailed and asked to wear an Axivity AX3 accelerometer (Newcastle upon Tyne, UK) on their dominant wrist for 24-h/day for seven days. The AX3 accelerometers were initialized to collect data with a sampling frequency of 100 Hz and a dynamic range of ±8 g. Participants returned the devices by mail and the data were calibrated and non-wear identified according to standard procedures [17] As described previously, acceleration signals were calibrated to gravity [18]. Data were combined into 5 s epochs representing an average vector magnitude. Non-wear time was identified as consecutive stationary episodes ≥60 min in which all three axes had standard deviation <13 mg [19]. MVPA was quantified by summing the number of 5-second epochs where the mean acceleration exceeded a threshold of >100 mg [20,21]. Individuals with <3 days of wear time or those that did not pass the data quality criteria were excluded [16,18].

### 2.6 Statistical analyses

For the cluster analysis for the first research question, the sample was stratified into two clusters (see above for details on the procedures). Then, each cluster was stratified into four quartiles of MVPA where the less physically active ‘Quartile 1′ was used as reference group. Quartiles of MVPA were also used for answering the second research question pertaining to individuals with AF undergoing a rhythm control procedure. For both aims, analysis was repeated with MVPA as a continuous variable in dose-response analyses.

Baseline characteristics were reported as median [interquartile range, IQR]. An independent *t*-test was used to compare differences between two groups. For comparisons among the four groups, a one-way analysis of variance (ANOVA) was conducted. In the case of a significant one-way interaction, post hoc independent *t*-tests were used to determine differences. To answer both research questions, we assess associations between accelerometer-derived MVPA and MACE, for each cluster, using Cox proportional hazards models, which were fitted with MACE as outcome and Quartiles of MVPA as exposure. MACE outcomes were censored with follow-up as 6-years or the first event following AF diagnosis (primary analysis) or AF procedure (subgroup analysis). Subsequently, unadjusted and adjusted multivariate survival analyses were undertaken. Adjusted models included age, sex, sedentary time, and cardiovascular risk factors for AF (HF, heart disease, T2D, BMI, smoking history, and hypertension) [22]. The same analysis was performed for the sensitivity analysis in a subgroup of patients who did not undergo AF procedure. To answer research question 1, we compared the 95 % Confidence Intervals (CI) for the (adjusted) Hazard Ratios between the ‘high risk’ and ‘low risk’ clusters. Linearity was assessed using the Wald- test and subsequent dose-response curves were produced using Poisson regression models with a restricted cubic spline fitted including knots at 10, 50, 90 percentiles. This was performed to better fit non-linear relationships (as expected with MVPA data) across the MVPA range of 0 to 300 min. From the Poisson-dose response curves, the minimal (first significant value versus the first minimal MVPA dose) and optimal MVPA dose (first non-significant value versus the previous 10 %, i.e.*,* 30 min as maximum benefit for least effort) to provide statistical representation of changes in the dose-response curve [23]. The risk of MACE for both minimum and optimum dose was then investigated compared to the reference value via Poisson regression producing incidence rate ratios (IRR). All statistical analyses and figures were performed and created in R version 4.3.1 (PBC, Boston, MA, USA) [24]. Statistical analyses were performed using the following packages: survival [25] and survminer [26]. Figures were created using the package ggplot2 [27]. Dose-response relationships analyses was performed using RiskRegression [28] and Splines2 [29] packages. P-values <0.05 were considered statistically significant.

## 3 Results

From the UK Biobank, 36,320 individuals with a diagnosis of AF were identified. Subsequently, the majority of the individuals (*n* = 30,650) were excluded due to insufficient accelerometer wear time (<3 days), insufficient data quality, or no accelerometery data. Then, we excluded *n* = 812 individuals for unavailability for the 30 variables, resulting in 4858 individuals with AF and validated accelerometer derived MVPA data (Figure S2). Median age was 63 [58–66] years and 37 % were female (Table S1). Median accelerometer wear time was 6.9 days [6.7–7.0].

### 3.1 Dose-response MVPA and MACE: impact of cardiovascular risk profile

#### 3.1.1 Cluster analysis

Two clusters were identified as phenotypically distinct and appropriately sized for the analysis (Figure S3). Individuals in Cluster 1 (*n* = 2275; ‘low risk’) were younger adults with a high proportion of females, cardiovascular risk factors, and comorbidities compared to those in the Cluster 2 (*n* = 2583, ‘high risk’, Table S1). In total, 764 MACEs occurred within the 6-year follow-up. In the ‘low risk’ Cluster, a total of 156 MACEs (7 %) occurred over a median follow-up of 72 months, including 22 strokes, 63 HF, 31 MI, 69 heart diseases, and 16 cardiac arrests. Among them, 31 individuals experienced more than one MACE. In the ‘high risk’ Cluster, a total of 608 MACEs (24 %) occurred, including 122 stroke, 263 HF, 128 MI, 201 heart diseases, and 28 cardiac arrests. Among them, 115 individuals experienced more than one MACE. Cox models demonstrated a significantly higher risk of MACE in the ‘high risk’ Cluster compared to the ‘low risk’ Cluster as a reference (HR 3.81, 95 %CI 3.19–4.55, *p* < 0.001, Figure S4).

#### 3.1.2 MVPA and MACE within clusters

**Fig. 1:**
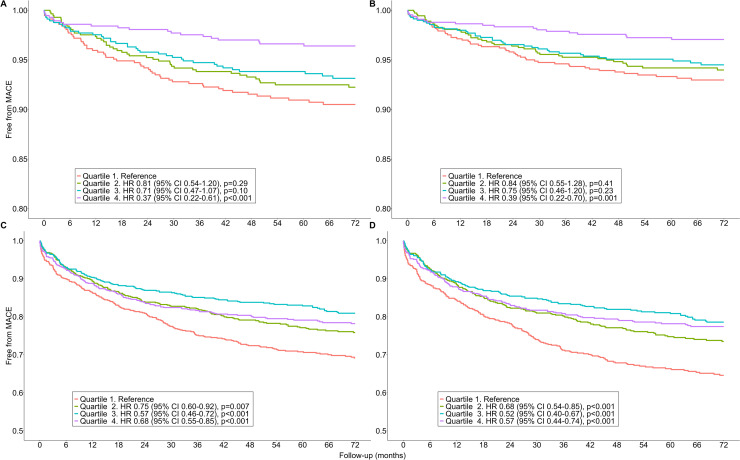
Survival analyses from major adverse cardiovascular events risks in individuals with atrial fibrillation of the ‘low risk’ cluster 1 (A-B, *n* = 2275) and the ‘high risk’ cluster 2 (C-D, *n* = 2583) according to physical activity volume (details). Unadjusted analyses were presented under A and C, whilst the adjusted analysis (B and D) was corrected for age, sex, ethnicity sedentarity, body mass index, alcohol and tobacco use, and cardiovascular risk factors for atrial fibrillation. MACE: major adverse cardiovascular events. P-values refer to the comparison to the reference group (i.e., Quartile 1’).

In the 'low risk' Cluster, the less physically active group 'Quartile 1′ had a median MVPA of 56 [46, 69] min/week (Table 1). The more physically active group ('Quartile 4′: median MVPA 187 [157, 199] min/week) showed lower MACE incidence compared to the reference group 'Quartile 1′ (HR 0.37, 95 %CI 0.22–0.61, *p* < 0.001; Fig. 1A). After adjusting for potential confounders, this relationship remained, with higher activity levels showing 61 % lower MACE incidence compared to 'Quartile 1′ (HR 0.39, 95 % CI 0.22–0.70, *p* = 0.001; Fig. 1B).

**Table 1:** Characteristics of the individuals with atrial fibrillation in ‘low risk’ cluster 1 according to their physical activity volume.

	Quartile 1 (*n* = 569)	Quartile 2 (*n* = 569)	Quartile 3 (*n* = 569)	Quartile 4 (*n* = 568)	P-values
**Characteristics**					
MACE, n ( %)	53 (9)	45 (8)	38 (7)	20 (4)	**<0.001**
Age, years	61 [58, 66]^a,b,c^	60 [56, 65]^b,c^	59 [54, 64]^c^	58 [53, 63]	**<0.001**
Female sex, n ( %)	200 (35)	184 (32)	177 (31)	215 (38)	0.08
White background, n ( %)	559 (98)	559 (98)	559 (98)	559 (98)	>0.99
BMI, kg/m^2^	29.5 [25.4, 32.2]^a,b,c^	28.2 [24.9, 30.7]^b,c^	26.9 [24.2, 29]^c^	26 [23.3, 28]	**<0.001**
DBP, mmHg	84 [77, 91]^b,c^	83 [76, 90]^c^	82 [75, 89]	81 [74, 89]	**<0.001**
SBP, mmHg	144 [130, 156]^b,c^	142 [130, 153]^c^	141 [127, 154]	138 [125, 150]	**<0.001**
eGFR, mL/min/1.73m^2^	96.3 [90.9, 102.6]^a,b,c^	99.8 [95.7, 106]	101.5 [97.5, 107.5]	99.8 [96.8, 106.3]	0.08
Cystatin C, mg/L	1.0 [0.9, 1.0]^a,b,c^	0.9 [0.8, 1.0]^c^	0.9 [0.8, 1.0]^c^	0.9 [0.8, 1.0]	**<0.001**
Haematocrit, %	42.1 [39.9, 44.5]^c^	42.3 [40.3, 44.5]^a,b,c,d,e,f^	42.2 [40, 44.3]	41.7 [39.6, 43.8]	**0.02**
Gamma-glutamyl transferase, U/L	48.2 [22.4, 53.5]^b,c^	45.6 [21.7, 50]^c^	42 [20.8, 47.7]	38.1 [18.6, 43.9]	**0.002**
HDL, mmol/L	1.3 [1, 1.5]^c^	1.3 [1.1, 1.5]^c^	1.3 [1.1, 1.5]^c^	1.4 [1.1, 1.6]	**0.02**
LDL, mmol/L	1.6 [1.3, 1.9]	1.6 [1.3, 1.9]	1.7 [1.4, 2]	1.7 [1.4, 1.9]	0.43
Alcohol - daily use, n ( %)	227 (40)	197 (35)	215 (38)	204 (36)	0.48
Tobacco Current, n ( %)	55 (10)^b,c^	39 (7)	32 (6)	32 (6)	**0.03**
Previous, n ( %)	243 (43)	225 (40)	254 (45)	248 (44)	0.59
Never, n ( %)	271 (48)	302 (53)	282 (50)	285 (50)	0.63
**Medication**					
Anti-hypertensive medication use, n ( %)	198 (35)^a,b,c^	153 (27)^b,c^	122 (21)	100 (18)	**<0.001**
Cholesterol lowering medication use, n ( %)	170 (30)^a,b,c^	150 (26)^b,c^	110 (19)	98 (17)	**<0.001**
Insulin use, n ( %)	4 (0.7)	4 (0.7)	2 (0.4)	1 (0.2)	0.48
Warfarin use, n ( %)	53 (9)	53 (9)	44 (8)	38 (7)	0.33
**Cardiovascular risk factors**					
Heart failure, n ( %)	16 (3)	15 (3)	12 (2)	10 (2)	0.63
Type 2 diabetes, n ( %)	39 (7)^a,b,c^	31 (5)^b,c^	15 (3)	6 (1)	**<0.001**
Hypertension, n ( %)	1 (0.2)	1 (0.2)	0 (0)	0 (0)	0.57
**Comorbidities**					
History of ischemic heart disease, n ( %)	90 (16)^b,c^	71 (12)	63 (11)	52 (9)	**0.01**
History of peripheral artery disease, n ( %)	1 (0.2)	2 (0.4)	0 (0)	0 (0)	0.30
History of hypothyroidism, n ( %)	29 (5)	16 (3)	18 (3)	22 (4)	0.20
History of hyperthyroidism, n ( %)	15 (3)	9 (2)	9 (2)	6 (1)	0.22
GI bleeding history, n ( %)	27 (5)	24 (4)	19 (3)	23 (4)	0.70
History of stroke, n ( %)	19 (3)	28 (5)	21 (4)	20 (4)	0.52
Intraventricular conduction: LBBB, n ( %)	11 (2)	16 (3)^c^	15 (3)^c^	4 (0.7)	0.05
Electrocardiographic evidence of LVH, n ( %)	12 (2)	10 (2)	5 (0.9)	13 (2)	0.28
Dementia, n ( %)	8 (1)^b^	5 (0.9)	1 (0.2)	0 (0)	**0.008**
History of CABG, n ( %)	5 (0.9)	2 (0.4)	2 (0.4)	4 (0.7)	0.56
History of PCI, n ( %)	0 (0)	2 (0.4)	3 (0.5)	2 (0.4)	0.44
Cardioversion, n ( %)	149 (26)	130 (23)	132 (23)	131 (23)	0.61
Catheter ablation, n ( %)	100 (18)^c^	109 (19)	123 (22)	134 (24)	0.12
Pacemaker, n ( %)	0 (0)	0 (0)	1 (0.2)	0 (0)	0.39
Renal replacement therapy/kidney transplantation, n ( %)	0 (0)	0 (0)	0 (0)	0 (0)	–
Anaemia, n ( %)	569 (100)	569 (100)	569 (100)	568 (100)	>0.99
Malignancy, n ( %)	139 (24)	143 (25)	130 (23)	120 (21)	0.50
Sleep apnoea, n ( %)	47 (8)	27 (5)	16 (3)	20 (4)	**<0.001**
COPD, n ( %)	25 (4)	21 (4)	9 (2)	10 (2)	**0.008**
CKD, n ( %)	33 (6)	15 (3)	12 (2)	17 (3)	**0.003**
**Physical activity**					
MVPA, min/week	56 [46, 69]^a,b,c^	90 [82, 98]^b,c^	124 [115, 132]^c^	187 [157, 199]	**<0.001**
LPA, min/week	262 [220, 300]^a,b,c^	293 [252, 328]^b,c^	303 [264, 338]^c^	350 [282, 377]	**<0.001**
Sedentary, min/week	1175 [1106, 1204]^a,b,c^	1120 [1047, 1135]^b,c^	1067 [1005, 1093]^c^	1107 [952, 1097]	**<0.001**

Continuous variables are expressed as median [IQR]; dichotomous variables are expressed as number and percentage.

MACE : major adverse cardiovascular events; BMI: body mass index; DBP: diastolic blood pressure; SBP: systolic blood pressure; eGFR: estimated glomerular filtration rate; HDL: high-density lipoprotein; LDL: low-density lipoprotein; GI bleeding: gastro-intestinal bleeding; LBBB: left Bundle branch block; LVH: left ventricular hypertrophy; CABG: coronary artery bypass graft; PCI: percutaneous coronary intervention; COPD: chronic obstructive pulmonary disease; CKD: chronic kidney disease; MVPA: moderate-vigorous physical activity; LPA: light physical activity.

^a^: vs Quartile 2, *p* < 0.05; ^b^: vs Quartile 3, *p* < 0.05 ; ^c^: vs Quartile 4, *p* < 0.05.

In the 'high risk' Cluster, the less physically active group 'Quartile 1′ had a median MVPA of 42 [32, 54] min/week (Table 2). Higher activity levels in 'Quartile 2′ (median MVPA 167 [137, 183] min/week), 'Quartile 3′ (median MVPA 167 [137, 183] min/week), and 'Quartile 4′ (median MVPA 167 [137, 183] min/week) were associated with lower MACE incidence compared to the reference group 'Quartile 1′ ('Quartile 2′: HR 0.75, 95 %CI 0.60–0.92, *p* = 0.007; 'Quartile 3′: HR 0.57, 95 %CI 0.46–0.72, *p* < 0.001; and 'Quartile 4′: HR 0.68, 95 %CI 0.55–0.85, *p* < 0.001, respectively; Fig. 1C). These relationships between MVPA and MACE incidence in the 'high risk' Cluster persisted in the adjusted analysis (Fig. 1D).

#### 3.1.3 Dose-response analysis

**Fig. 2:**
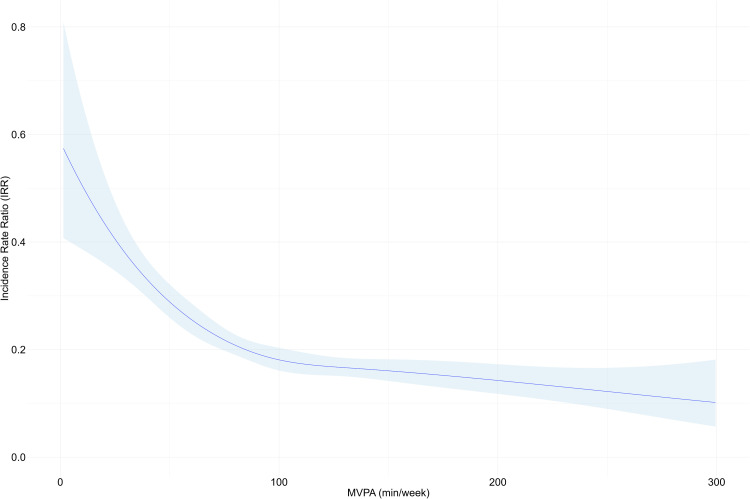
Poisson regression dose-response analysis between MVPA (min/week) and total count of MACE in all individuals with atrial fibrillation. Restricted cubic spline (fitted with three knots to account for non-linear relationship). MVPA: Moderate-to-vigorous physical activity, MACE; Major adverse cardiovascular events.

A non-linear relationship (Wald test, *p* < 0.001) between MVPA levels and MACE incidence (Fig. 2) was observed for individuals with AF. Using Poisson regression, a notable threshold was identified at 35 min/week of MVPA. Compared to the lowest MVPA levels ('reference dose'), this threshold corresponded to 65 % lower MACE incidence (IRR 0.35, 95 % CI 0.31–0.39, *p* < 0.001). A second notable threshold was identified at 103 min/week, which corresponded to 69 % lower MACE incidence compared to the reference levels (IRR 0.31, 95 % CI 0.22–0.45, *p* < 0.001).

**Table 2:** Characteristics of the individuals with atrial fibrillation in ‘high risk’ cluster 2 according to their physical activity volume.

	Quartile 1 (*n* = 646)	Quartile 2 (*n* = 646)	Quartile 3 (*n* = 646)	Quartile 4 (*n* = 645)	P-values
**Characteristics**					
MACE, n ( %)	196 (30)^a,b,c^	153 (24)^b^	120 (19)	139 (22)	**<0.001**
Age, years	64 [62, 68]^b,c^	64 [61, 67]^b,c^	63 [61, 66]^c^	62 [60, 66]	**<0.001**
Female sex, n ( %)	235 (36)^c^	230 (36)^c^	264 (41)	283 (44)	**0.006**
White background, n ( %)	638 (99)	633 (98)	633 (98)	628 (97)	0.994
BMI, kg/m^2^	30.4 [26.4, 33.3]^a,b,c^	28.7 [25.1, 31.2]^b,c^	27.7 [24.7, 30.2]^c^	26.8 [24, 29.2]	**<0.001**
DBP, mmHg	83 [76, 90]	82 [75, 89]	82 [75, 88]	82 [74, 89]	0.684
SBP, mmHg	148 [132, 162]^a,b,c^	146 [132, 158]	144 [132, 157]	144 [130, 156]	**0.005**
eGFR, mL/min/1.73m^2^	92.7 [89, 99.6]^c^	93.7 [91.3, 100.8]^c^	94.8 [92.4, 100.5]	96.1 [92.7, 102.9]	0.19
Cystatin C, mg/L	1.1 [0.9, 1.1]^a,b,c^	1 [0.9, 1.1]^b,c^	1 [0.9, 1]^c^	0.9 [0.8, 1]	**<0.001**
Haematocrit, %	41.6 [39.4, 44.2]	41.9 [39.6, 44.2]^c^	41.7 [39.3, 44]	41.4 [39.3, 43.7]	0.06
Gamma-glutamyl transferase, U/L	44.8 [22.4, 49.6]^b,c^	43.2 [20.8, 46.6]^c^	38.7 [20.3, 42]	35 [19.6, 38.6]	**<0.001**
HDL, mmol/L	1.2 [1.0, 1.4]	1.2 [1.0, 1.4]	1.3 [1.0, 1.4]	1.3 [1.0, 1.4]	0.30
LDL, mmol/L	1.5 [1.2, 1.8]^c^	1.6 [1.3, 1.9]	1.6 [1.3, 1.9]	1.7 [1.4, 1.9]	0.15
Alcohol - daily use, n ( %)	88 (14)	79 (12)	94 (15)^c^	68 (11)	0.20
Tobacco Current, n ( %)	47 (7)^b,c^	35 (5)	20 (3)	21 (3)	**0.001**
Previous, n ( %)	340 (53)^b,c^	315 (49)^c^	297 (46)	273 (42)	**<0.05**
Never, n ( %)	256 (40)^a,b,c^	292 (45)^c^	329 (51)	348 (54)	**<0.001**
**Medication**					
Anti-hypertensive medication use, n ( %)	311 (48)^a,b,c^	261 (40)^b,c^	251 (39)^c^	171 (27)	**<0.001**
Cholesterol lowering medication use, n ( %)	274 (42)^a,b,c^	233 (36)^b,c^	204 (32)^c^	157 (24)	**<0.001**
Insulin use, n ( %)	17 (3)^b,c^	13 (2)	6 (0.9)	4 (0.6)	**0.01**
Warfarin use, n ( %)	72 (11)	68 (11)	62 (10)	51 (8)	0.27
**Cardiovascular risk factors**					
Heart failure, n ( %)	118 (18)^a,b,c^	60 (9)	67 (10)	57 (9)	**<0.001**
Type 2 diabetes, n ( %)	152 (24)^a,b,c^	86 (13)^b,c^	48 (7)	35 (5)	**<0.001**
Hypertension, n ( %)	0 (0)	2 (0.3)	2 (0.3)	1 (0.2)	0.53
**Comorbidities**					
History of ischemic heart disease, n ( %)	355 (55)^a,b,c^	299 (46)^b,c^	257 (40)	241 (37)	**<0.001**
History of peripheral artery disease, n ( %)	74 (11)^a,b,c^	48 (7)^b,c^	28 (4)	25 (4)	**<0.001**
History of hypothyroidism, n ( %)	104 (16)^c^	97 (15)	85 (13)	76 (12)	0.16
History of hyperthyroidism, n ( %)	23 (4)^c^	21 (3)^c^	13 (2)	9 (1)	**<0.05**
GI bleeding history, n ( %)	69 (11)^a,b,c^	46 (7)	31 (5)	29 (4)	**<0.001**
History of stroke, n ( %)	211 (33)^a,b,c^	135 (21)^c^	124 (19)	99 (15)	**<0.001**
Intraventricular conduction: LBBB, n ( %)	56 (9)^b,c^	38 (6)	33 (5)	27 (4)	**0.007**
Electrocardiographic evidence of LVH, n ( %)	43 (7)	35 (5)	30 (5)	45 (7)	0.28
Dementia, n ( %)	27 (4)^a^	10 (2)	14 (2)	14 (2)	**0.02**
History of CABG, n ( %)	79 (12)^b,c^	73 (11)^c^	54 (8)	50 (8)	**0.02**
History of PCI, n ( %)	31 (5) ^a,c^	15 (2)	23 (4)	16 (2)	0.05
Cardioversion, n ( %)	93 (14)	90 (14)	91 (14)	85 (13)	0.94
Catheter ablation, n ( %)	57 (9)	43 (7)^b,c^	69 (11)	73 (11)	**0.03**
Pacemaker, n ( %)	4 (0.6)	3 (0.5)	11 (2)	3 (0.5)	**0.04**
Renal replacement therapy/kidney transplantation, n ( %)	9 (1)	2 (0.3)	2 (0.3)	4 (0.6)	0.05
Anaemia, n ( %)	646 (100)	646 (100)	646 (100)	645 (100)	>0.99
Malignancy, n ( %)	143 (22)	132 (20)	114 (18)	101 (16)	**0.04**
Sleep apnoea, n ( %)	51 (8)	28 (4)	14 (2)	15 (2)	**<0.001**
COPD, n ( %)	122 (19)	72 (11)	61 (9)	60 (9)	**<0.001**
CKD, n ( %)	188 (29)^c^	111 (17)^c^	98 (15)	79 (12)	**<0.001**
**Physical activity**					
MVPA, min/week	42 [32, 54]^a,b,c^	77 [70, 84]^b,c^	107 [99, 114]^c^	167 [137, 183]	**<0.001**
LPA, min/week	250 [207, 290]^a,b,c^	287 [245, 324]^b,c^	307 [262, 342]^c^	344 [290, 376]	**<0.001**
Sedentary, min/week	119 [1128, 1237]^a,b,c^	1130 [1065, 1157]^b,c^	1095 [1022, 1115]^c^	1091 [959, 1104]	**<0.001**

Continuous variables are expressed as median [IQR]; dichotomous variables are expressed as number and percentage.

MACE : major adverse cardiovascular events; BMI: body mass index; DBP: diastolic blood pressure; SBP: systolic blood pressure; eGFR: estimated glomerular filtration rate; HDL: high-density lipoprotein; LDL: low-density lipoprotein; GI bleeding: gastro-intestinal bleeding; LBBB: left Bundle branch block; LVH: left ventricular hypertrophy; CABG: coronary artery bypass graft; PCI: percutaneous coronary intervention; COPD: chronic obstructive pulmonary disease; CKD: chronic kidney disease; MVPA: moderate-vigorous physical activity; LPA: light physical activity.

^a^: vs Quartile 2, *p* < 0.05; ^b^: vs Quartile 3, *p* < 0.05 ; ^c^: vs Quartile 4, *p* < 0.05.

### 3.2 Dose-response MVPA and MACE: impact of rhythm control procedure

**Fig. 3:**
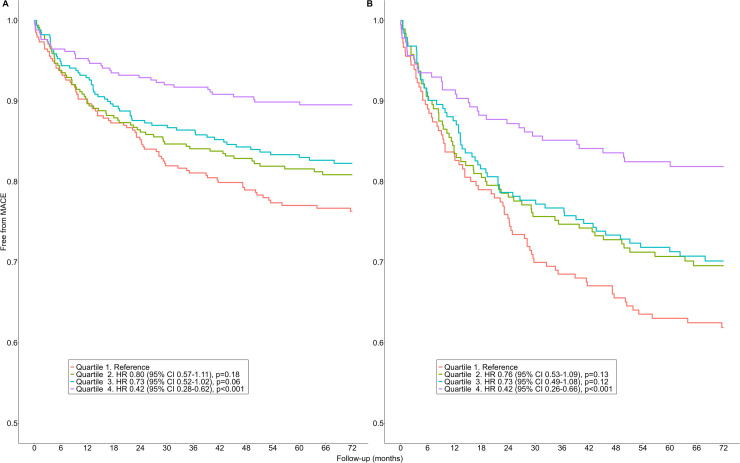
Survival analyses from major adverse cardiovascular events risks in individuals with atrial fibrillation undergoing rhythm control procedures according to physical activity volume. Unadjusted (A) and adjusted (B) for age, sex, ethnicity sedentarity, body mass index, alcohol and tobacco use, and cardiovascular risk factors for atrial fibrillation. MACE: major adverse cardiovascular events. P-values refer to the comparison to the reference group (i.e., ‘Quartile 1′).

Among the individuals with AF, 1354 (28 %) received a rhythm control procedure (Table 3). When stratified by quartiles of MVPA, MACE incidence varied across activity levels, with events occurring in 80 (24 %), 64 (19 %), 59 (17 %), and 35 (10 %) participants in 'Quartile 1′, 'Quartile 2′, 'Quartile 3′, and 'Quartile 4′, respectively. Compared to the reference 'Quartile 1′ group (median MVPA 54 [41, 64] min/week), those with higher physical activity levels ('Quartile 4′, median MVPA 168 [151, 194] min/week) showed lower MACE incidence (HR 0.42, 95 %CI 0.28–0.62, *p* < 0.001, Fig. 3A). This relationship persisted in the adjusted analysis (HR 0.42, 95 %CI 0.26–0.66, *p* < 0.001, Fig. 3B).

**Table 3:** Characteristics of the individuals with atrial fibrillation undergoing rhythm control procedure according to their physical activity volume.

	Overall (*n* = 1354)	Quartile 1 (*n* = 339)	Quartile 2 (*n* = 339)	Quartile 3 (*n* = 338)	Quartile 4 (*n* = 338)	P-values
**Characteristics**						
MACE, n ( %)	238 (18)	80 (24)^a,b,c^	64 (19)^c^	59 (17)^c^	35 (10)	**<0.001**
Age, years	60 [57, 65]	62 [59, 66]^a,b,c^	61 [58, 65]^b,c^	59 [55, 64]	58 [54, 63]	**<0.001**
Female sex, n ( %)	390 (29)	91 (27)	103 (30)	93 (28)	103 (30)	0.62
White background, n ( %)	1328 (98)	332 (98)	335 (99)	328 (97)	333 (99)	0.99
BMI, kg/m^2^	27.9 [24.6, 30.4]	30.1 [26.3, 33.2]^a,b,c^	28.2 [24.6, 30.9]^b,c^	27.3 [24.5, 29.6]^c^	26.2 [23.6, 28.1]	**<0.001**
DBP, mmHg	83 [76, 90]	84 [76, 90]	84 [76, 90]	83 [75, 90]	82 [74, 90]	0.46
SBP, mmHg	143 [128, 155]	145 [130, 156]^c^	142 [128, 153]	142 [129, 155]	140 [125, 153]	0.06
eGFR, mL/min/1.73m^2^	97.5 [94.3, 103.6]	96.2 [90.9, 101.4]	96.3 [93.4, 103.3]	99.6 [96.5, 105.9]	97.8 [95.9, 104.4]	0.96
Cystatin C, mg/L	1.0 [0.9, 1.0]	1.0 [0.9, 1.1]^a,b,c^	1 0.0[0.9, 1.0]^c^	0.9 [0.9, 1.0]	0.9 [0.8, 1]	**<0.001**
Haematocrit, %	42.3 [40.2, 44.6]	42.2 [40, 44.7]	42.4 [40, 44.7]	42.4 [40.3, 44.7]	42 [40.1, 44.4]	0.36
Gamma-glutamyl transferase, U/L	43.1 [21.3, 47.6]	50.6 [22.9, 55.8]^a,b,c^	41.8 [22.1, 48.7]	40 [21.3, 44.3]	40.4 [19.3, 42.7]	**0.009**
HDL, mmol/L	1.3 [1, 1.4]	1.2 [1, 1.4]^c^	1.2 [1, 1.4]^c^	1.3 [1, 1.4]	1.3 [1.1, 1.5]	**0.04**
LDL, mmol/L	1.6 [1.3, 1.9]	1.6 [1.3, 1.8]	1.6 [1.3, 1.8]	1.6 [1.5, 1.8]	1.7 [1.4, 1.9]	0.37
Alcohol - daily use, n ( %)	359 (27)	80 (24)^c^	83 (24)^c^	88 (26)	108 (32)	0.15
Tobacco						
Current, n ( %)	82 (6)	25 (7)	21 (6)	18 (5)	18 (5)	0.66
Previous, n ( %)	609 (45)	169 (50)^b^	149 (44)	138 (41)	153 (45)	0.36
Never, n ( %)	661 (49)	144 (42)^b^	169 (50)	181 (54)	167 (49)	0.23
**Medication**						
Anti-hypertensive medication use, n ( %)	413 (31)	139 (41)^a,b,c^	105 (31)^c^	100 (30)^c^	69 (20)	**<0.001**
Cholesterol lowering medication use, n ( %)	352 (26)	128 (38)^a,b,c^	91 (27)^c^	73 (22)	60 (18)	**<0.001**
Insulin use, n ( %)	10 (0.7)	7 (2)	2 (0.6)	0 (0)	1 (0.3)	**0.009**
Warfarin use, n ( %)	201 (15)	69 (20)^b,c^	52 (15)^c^	47 (14)	33 (10)	**0.004**
**Cardiovascular risk factors**						
Heart failure, n ( %)	104 (8)	45 (13)^a,b,c^	20 (6)	19 (6)	20 (6)	**<0.001**
Type 2 diabetes, n ( %)	89 (7)	44 (13)^a,b,c^	22 (6)^c^	15 (4)	8 (2)	**<0.001**
Hypertension, n ( %)	4 (0.3)	1 (0.3)	3 (0.9)	0 (0)	0 (0)	0.11
**Comorbidities**						
History of ischemic heart disease, n ( %)	407 (30)	143 (42)^a,b,c^	100 (29)^c^	99 (29)^c^	65 (19)	**<0.001**
History of peripheral artery disease, n ( %)	46 (3)	24 (7)^a,b,c^	12 (4)^c^	7 (2)	3 (0.9)	**<0.001**
History of hypothyroidism, n ( %)	98 (7)	29 (9)	20 (6)	30 (9)	19 (6)	0.25
History of hyperthyroidism, n ( %)	33 (2)	14 (4)^b^	8 (2)	4 (1)	7 (2)	0.09
GI bleeding history, n ( %)	69 (5)	29 (9)^a^	11 (3)	16 (5)	13 (4)	**0.01**
History of stroke, n ( %)	166 (12)	63 (19)^a,b,c^	42 (12)	31 (9)	30 (9)	**<0.001**
Intraventricular conduction: LBBB, n ( %)	60 (4 %)	25 (7)^b,c^	14 (4)	12 (4)	9 (3)	**0.02**
Electrocardiographic evidence of LVH, n ( %)	89 (7 %)	31 (9)	18 (5)	19 (6)	21 (6)	0.19
Dementia, n ( %)	14 (1 %)	6 (2)	5 (1)	0 (0)	3 (0.9)	0.11
History of CABG, n ( %)	49 (4 %)	21 (6)^c^	12 (4)	12 (4)	4 (1)	**0.008**
History of PCI, n ( %)	15 (1 %)	3 (0.9)	2 (0.6)	7 (2)	3 (0.9)	0.27
Cardioversion, n ( %)	901 (67 %)	247 (73)^b,c^	233 (69)^c^	220 (65)	201 (59)	0.17
Catheter ablation, n ( %)	708 (52 %)	157 (46)^b,c^	158 (47)^b,c^	192 (57)	201 (59)	**0.03**
Pacemaker, n ( %)	5 (0.4 %)	2 (0.6)	1 (0.3)	2 (0.6)	0 (0)	0.53
Renal replacement therapy/kidney transplantation, n ( %)	2 (0.1 %)	1 (0.3)	0 (0)	0 (0)	1 (0.3)	0.57
Anaemia, n ( %)	1354 (100 %)	339 (100)	339 (100)	338 (100)	338 (100)	>0.99
Malignancy, n ( %)	74 (22 %)	74 (22)	68 (20)	55 (16)	60 (18)	0.35
Sleep apnoea, n ( %)	69 (5 %)	26 (8)^a,b,c^	25 (7)^b,c^	9 (3)	9 (3)	**0.001**
COPD, n ( %)	89 (7 %)	31 (9) ^b,c^	28 (8)	14 (4)	16 (5)	**0.02**
CKD, n ( %)	123 (9 %)	49 (14)^a,b,c^	26 (8)	26 (8)	22 (7)	**0.002**
**Physical activity**						
MVPA, min/week	103 [71, 137]	54 [41, 64]^a,b,c^	86 [79, 94]^b,c^	119 [111, 129]^c^	168 [151, 194]	**<0.001**
LPA, min/week	285 [247, 329]	252 [211, 288]^a,b,c^	279 [248, 320]^b,c^	298 [262, 337]^c^	315 [269, 364]	**<0.001**
Sedentary, min/week	1088 [1027, 1159]	1161 [1116, 1221]^a,b,c^	1098 [1060, 1141]^b,c^	1053 [1005, 1095]^c^	1021 [959, 1093]	**<0.001**

Continuous variables are expressed as median [IQR]; dichotomous variables are expressed as number and percentage.

MACE : major adverse cardiovascular events; BMI: body mass index; DBP: diastolic blood pressure; SBP: systolic blood pressure; eGFR: estimated glomerular filtration rate; HDL: high-density lipoprotein; LDL: low-density lipoprotein; GI bleeding: gastro-intestinal bleeding; LBBB: left Bundle branch block; LVH: left ventricular hypertrophy; CABG: coronary artery bypass graft; PCI: percutaneous coronary intervention; COPD: chronic obstructive pulmonary disease; CKD: chronic kidney disease; MVPA: moderate-vigorous physical activity; LPA: light physical activity.

^a^: vs Quartile 2, *p* < 0.05; ^b^: vs Quartile 3, *p* < 0.05 ; ^c^: vs Quartile 4, *p* < 0.05.

### 3.3 Senssitive analysis

As a sensitivity analysis, the primary analysis was repeated in a cohort of individuals with AF which excluded those who underwent a rhythm control procedure (*n* = 3504). Results remained largely consistent with the primary analysis. Compared to the reference ‘Quartile 1′ group (median MVPA 49 [36, 59] min/week), being more physically active were associated with lower risks of MACEs (vs ‘Quartile 2′, median MVPA 82 [75, 89] min/week, HR 0.60, 95 % CI 0.47–0.75, *p* < 0.001; vs ‘Quartile 3′, median MVPA 112 [104, 122] min/week, HR 0.51, 95 % CI 0.40–0.65, *p* < 0.001; vs ‘Quartile 4’, median MVPA 162 [144, 191] min/week, HR 0.49, 95 % CI 0.38–0.62, *p* < 0.001). In adjusted analysis, these effects remained significant (Table S2, Figure S5).

## 4 Discussion

The aim of this study was to describe the relationship between MVPA and occurrence of major adverse cardiovascular events (MACE) for individuals with AF and to characterize these patterns across risk phenotypes and among those undergoing rhythm control procedures. We performed a hierarchical cluster analysis to identify a 'low risk' Cluster and a 'high risk' Cluster, with the high risk cluster showing a 3.81-fold higher MACE incidence compared to the low risk cluster. Both clusters exhibited a non-linear relationship between MVPA levels and MACE incidence, with individuals engaging in 187 min/week ('low risk' Cluster) and 167 min/week ('high risk' Cluster) of MVPA having 61 % and 43 % lower MACE incidence over 6-year follow-up, respectively, compared to those with lower activity levels. Our analysis identified notable MVPA thresholds at 35 min/week and 103 min/week, which were associated with 65 % and 69 % lower MACE incidence, respectively, across all participants with AF. Among those who underwent rhythm control procedures, individuals with higher activity levels (>168 min/week of MVPA) showed 58 % lower MACE incidence. Collectively, this study documents that the relationship between MVPA levels and MACE incidence follows similar patterns across different AF phenotypes, including those based on cardiovascular risk profiles and rhythm control treatment status. These findings provide objective documentation of physical activity patterns and their association with cardiovascular outcomes in people with AF.

Two recent prospective cohort studies demonstrated in individuals with AF, either using self-reported (∼1000 participants) [6] or objectively assessed physical activity data (∼2000 participants) [30], that higher physical activity levels were associated with lower long-term risk of cardiovascular disease and all-cause mortality. The results of the present study document a curvilinear relationship between MVPA levels and MACE incidence in individuals with AF. More importantly, we found that these associations follow similar patterns across different cardiovascular risk profiles, with both the 'low' and 'high risk' clusters showing lower MACE incidence with higher activity levels. Specifically, despite a 3.81-fold higher MACE incidence in the 'high risk' cluster, comparable patterns of association between MVPA and cardiovascular outcomes were observed in both clusters. This observation is relevant, as some studies have suggested that the relationship between physical activity and cardiovascular outcomes may differ in populations with established cardiovascular disease [10,31]. Taken together, our data indicate that the association between regular MVPA and MACE incidence in subjects with AF follows similar patterns across different cardiovascular risk profiles.

The use of rigorous accelerometer data also enabled the determination of specific MVPA thresholds that associate with better clinical outcomes. For the first time, we identify notable MVPA thresholds (35 min/week and 103 min/week) that could inform clinical guidelines and support individuals with AF in changing physical activity behavior patterns. Although our analytical approach to determine these thresholds has been used previously [23]^.^ a consistent method does not exist across studies [32]. It is important to note that the curvilinear relationship in our study indicates that the strongest association between physical activity and reduced MACE incidence occurs at the lower end of the physical activity spectrum (Fig. 3). In other words, the data suggest that for inactive individuals with AF, MVPA levels between 35 and 103 min/week show particularly strong associations with improved outcomes. These observations align with the non-linear relationships documented in general populations[33] and highlight that relatively modest activity levels show meaningful associations with cardiovascular outcomes for participants with AF.

We also sought to better understand whether the role of physical activity differed for participants who underwent rhythm control procedures. This seems especially relevant since studies support the clinical relevance of rhythm control interventions, including catheter ablation [34,35]. Although ablation and cardioversion may reduce the risk for adverse cardiovascular events, risk for MACE remains high [36,37]. It is therefore clinically relevant that our study revealed that engagement in MVPA also seems beneficial for individuals with AF who underwent a rhythm control procedure. Specifically, MACE incidence was 58 % lower in individuals who performed >168 min/week of MVPA compared to those who performed <54 min/week. Thus, while rhythm control procedures are associated with reduced MACE incidence, our data show that among patients receiving these procedures, those with higher MVPA levels demonstrated lower cardiovascular event rates.

The beneficial associations of higher physical activity and lower MACE in the present study raises questions on the potential mechanisms. First, AF is associated with many modifiable cardiovascular risk factors (e.g., BMI, blood pressure, inflammation, cholesterol), and overt cardiovascular conditions [38,39]. which may be altered following regular MVPA and subsequently contributing to the lower risk for MACE. Furthermore, it has been hypothesised that physical activity improves atrial health (e.g.*,* atrial size, fibrosis, inflammation) and vascular structure and function (e.g.*,* vessel diameter, wall thickness, endothelial function), which may translate to improved outcomes for mortality and morbidity [13,40,41]. Nonetheless, future studies are warranted to better understand the mechanisms explaining the beneficial impacts of physical activity on clinical outcomes for people with AF.

These findings have direct relevance for clinical practice. They demonstrate that MVPA is associated with reduced cardiovascular event risk in individuals with AF, regardless of comorbidity burden or phenotype. This suggests that clinicians can confidently recommend physical activity—even at modest levels (≥35 min/week)—to all AF patients, including those with complex health profiles. The clustering analysis reinforces that MVPA is beneficial across diverse AF presentations, simplifying clinical messaging and supporting guideline-based activity targets as both achievable and impactful in routine care.

*Strengths and limitations.* Strengths of our study include device-based physical activity monitoring, inclusion of a large cohort of individuals with AF, adopting rigorous hierarchical cluster analyses, and a 6-year follow-up. Some limitations should be considered. Despite the strong effects on MACE, our study provides no insight into patient reported outcomes such as AF severity and burden, quality of life, and mental wellbeing, however, these are well documented in a recent Cochrane review [7]. This cohort is UK-based, which could limit the generalisation to other populations including low-to-middle income countries. Physical activity was measured at baseline only, and we cannot rule out changes in physical activity that have occurred across time and how this may have affected our results. In addition, the UK Biobank cohort is a relatively healthy cohort, demonstrating a ‘healthy volunteer bias’ [42]. In the same way, the UK Biobank also had a very low response rate (∼5.5 %), and participants in our sample were subject to additional selection criteria due to accelerometer participation (∼45 %) and the requirement for complete covariate data. These factors should be considered when interpreting our findings. However, prior evidence indicates that such unrepresentativeness does not materially bias associations between lifestyle risk factors (including physical activity) and mortality [43]. Moreover, recent studiy that provides consistent support for our findings, helping contextualize plausibility [30]. To further contextualize generalisability, we have added a supplementary table comparing baseline characteristics of included versus excluded AF participants. Moreover, the clustering approach and MVPA threshold identification are sample-specific, which may limit reproducibility in other datasets. That is, machine-learning-based methods may provide more procise estimates and further research should leverage these updated metrics [44]. Finally, our secondary analysis defined the “rhythm control” subgroup by exposure to a procedure (catheter ablation or cardioversion), which does not necessarily reflect achievement or maintenance of durable rhythm control. Nevertheless, the consistency of associations in this subgroup supports the overall robustness of our findings regarding the benefits of MVPA.

## 5 Conclusion

Our main finding is that a non-linear relationship exists between moderate-to-vigorous physical activity levels and MACE incidence in people with AF across a 6-year follow-up. These patterns appear similar across different risk phenotypes and among patients undergoing rhythm control procedures. Specifically, engagement in >187 and 167-min/week of MVPA was associated with 61 % and 43 % lower MACE incidence in the 'low risk' and 'high risk' clusters, respectively. Moreover, due to the curvilinear relationship between MVPA and MACE, a notable threshold of MVPA associated with lower MACE incidence was identified at 35 min/week. Collectively, these findings document physical activity patterns and their association with cardiovascular outcomes in people with AF, and may inform future physical activity recommendations for this population.

## Data availability

The UK Biobank data that support the findings of this study can be accessed by researchers on application (https://www.ukbiobank.ac.uk/register-apply/).

## Funding

No funding

Central illustration

## CRediT authorship contribution statement

**Maxime Boidin:** Writing – review & editing, Writing – original draft, Validation, Software, Methodology, Formal analysis, Conceptualization. **Benjamin JR Buckley:** Writing – review & editing, Writing – original draft, Validation, Software, Methodology, Formal analysis, Conceptualization. **Gregory YH Lip:** Writing – review & editing, Writing – original draft, Validation, Supervision, Methodology, Formal analysis, Conceptualization. **Dick HJ Thijssen:** Writing – review & editing, Writing – original draft, Validation, Supervision, Methodology, Formal analysis, Conceptualization.

## Declaration of competing interest

The authors declare that they have no known competing financial interests or personal relationships that could have appeared to influence the work reported in this paper.

## Appendix Supplementary materials

Image, image 1

Image, image 2

Image, image 3

Image, image 4

Image, image 5

Image, application 6
