# Supplementary figures and images for: Impact of patient phenotype on the relationship between accelerometer-derived physical activity and cardiovascular events in atrial fibrillation
Source: Am J Prev Cardiol. 2025 Dec 5;25:101362. doi: 10.1016/j.ajpc.2025.101362 (PMC12743516; doi:10.1016/j.ajpc.2025.101362)

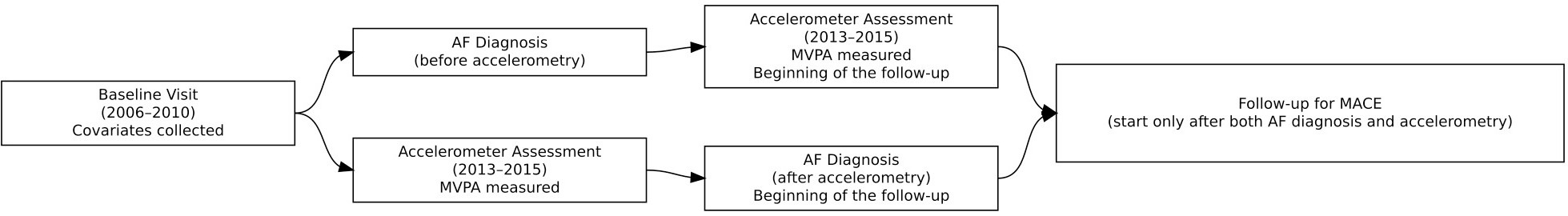

Supplement: Supplementary file 1 [file mmc1.jpg]

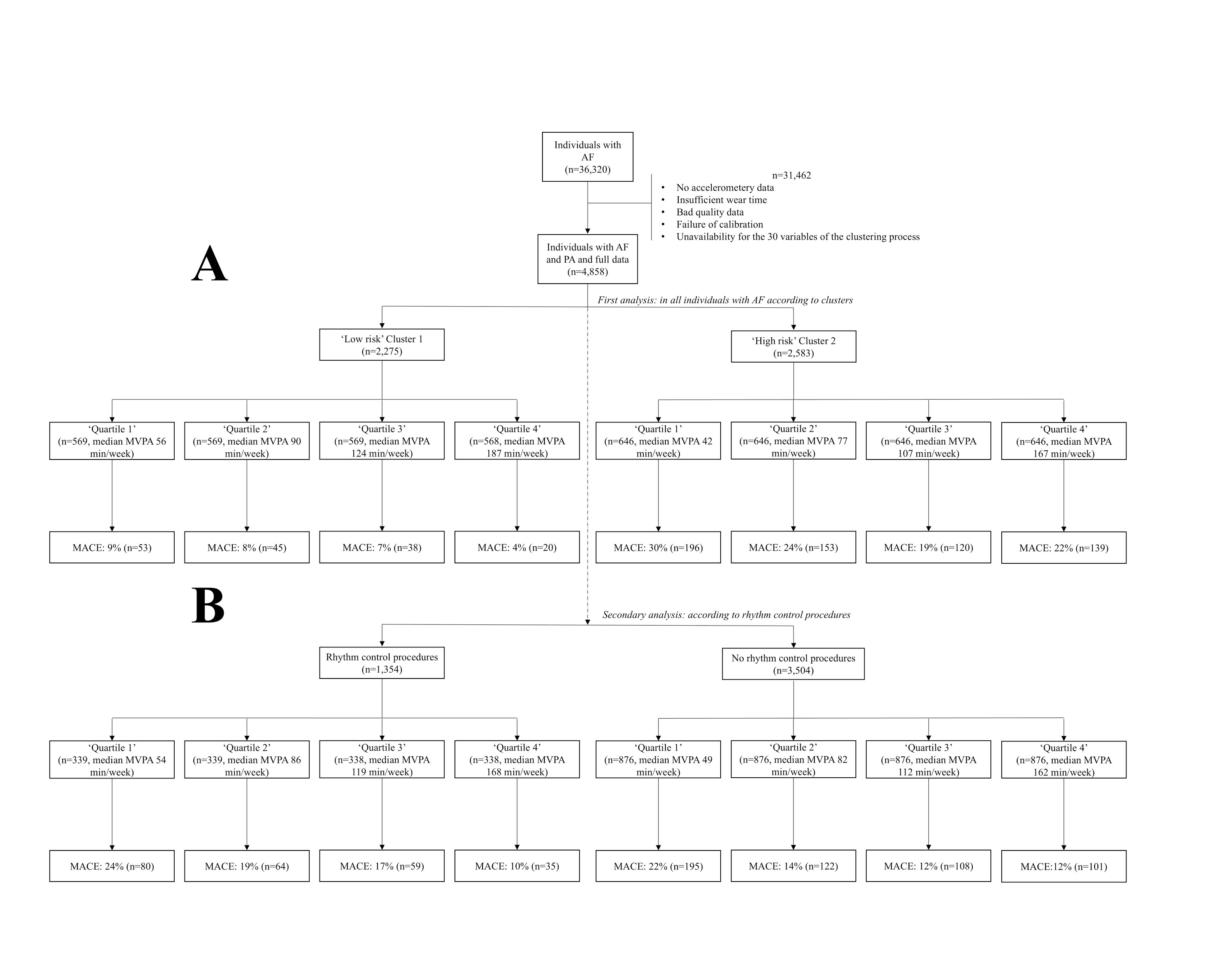

Supplement: Supplementary file 2 [file mmc2.jpg]

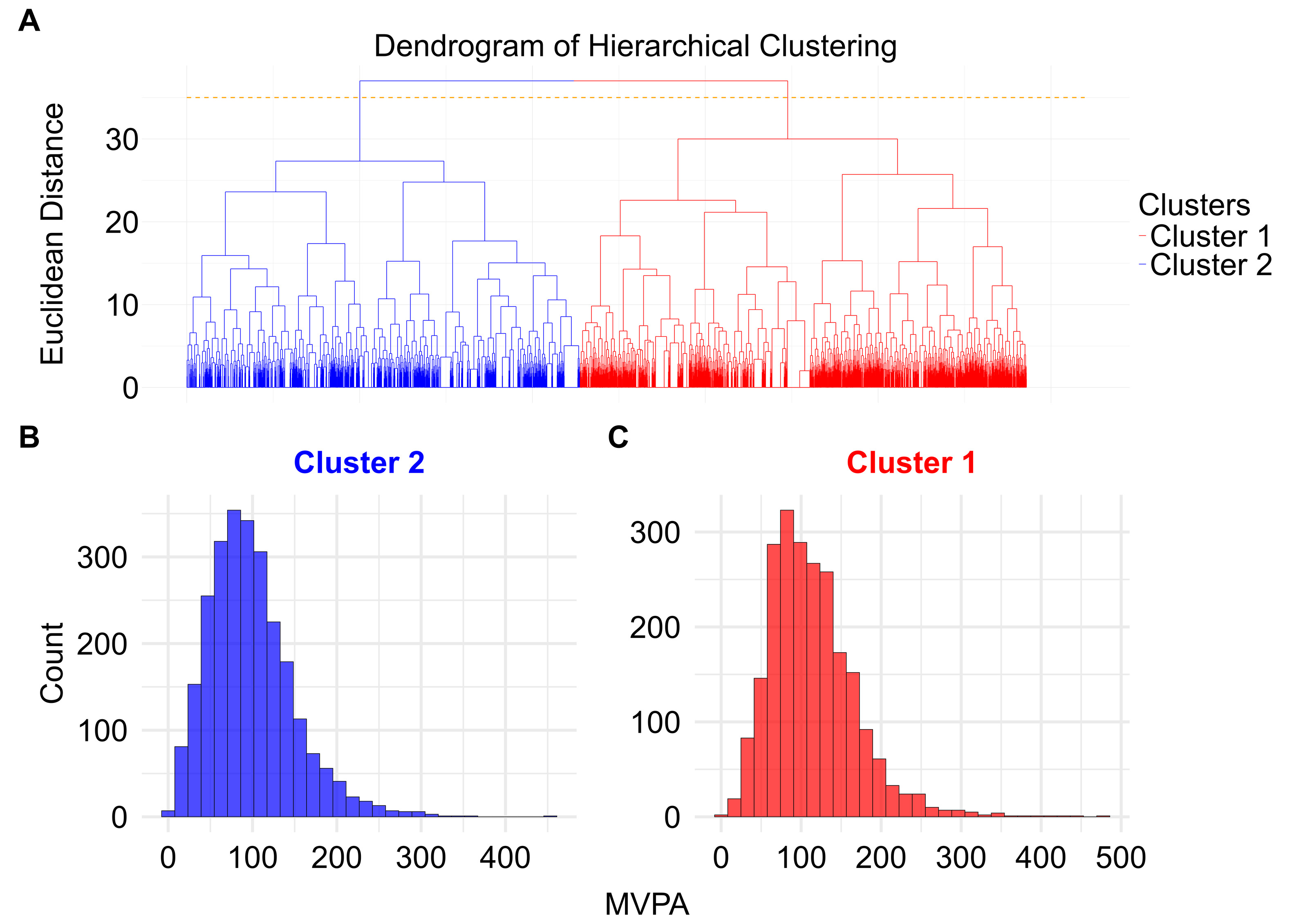

Supplement: Supplementary file 3 [file mmc3.jpg]

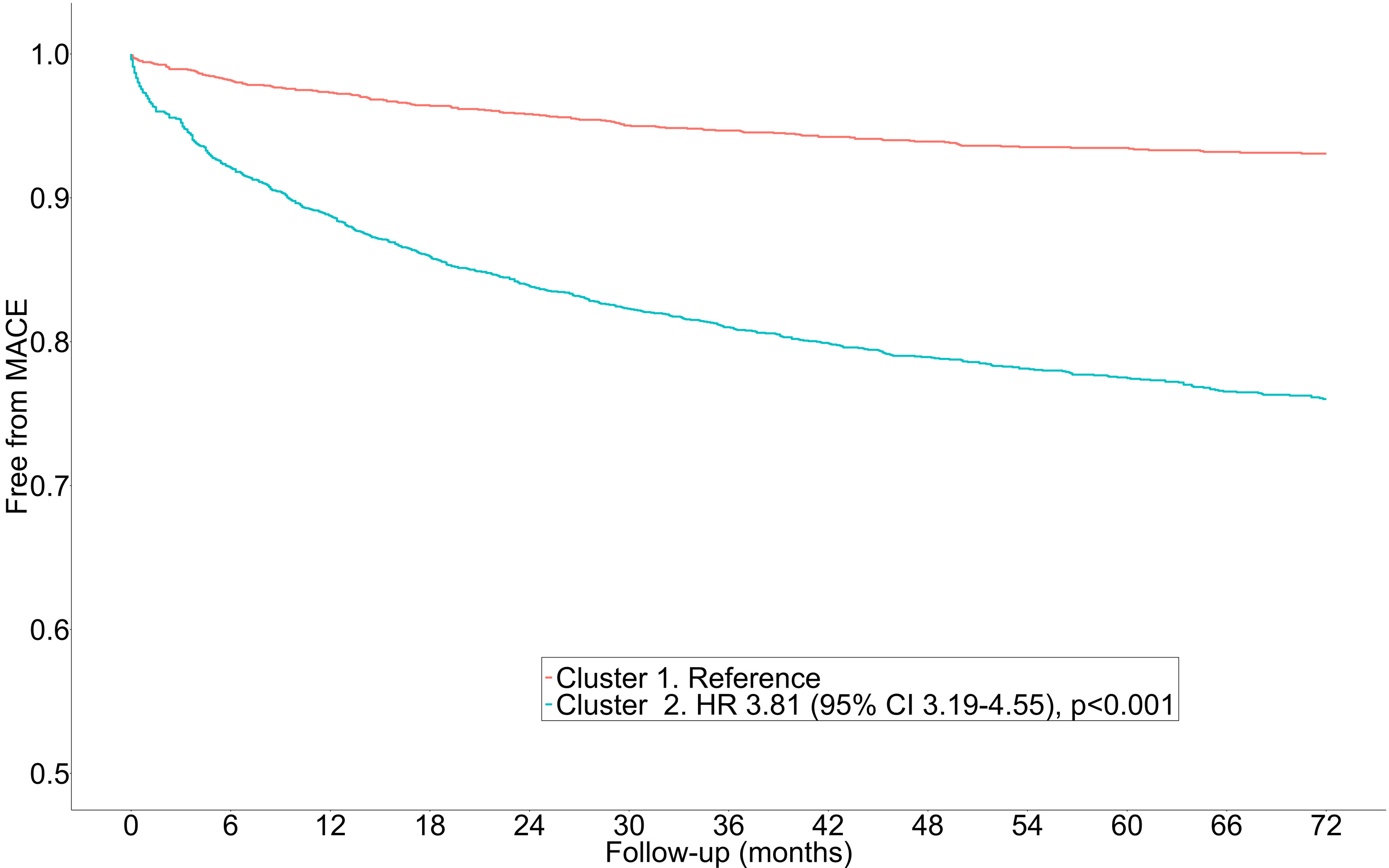

Supplement: Supplementary file 4 [file mmc4.jpg]

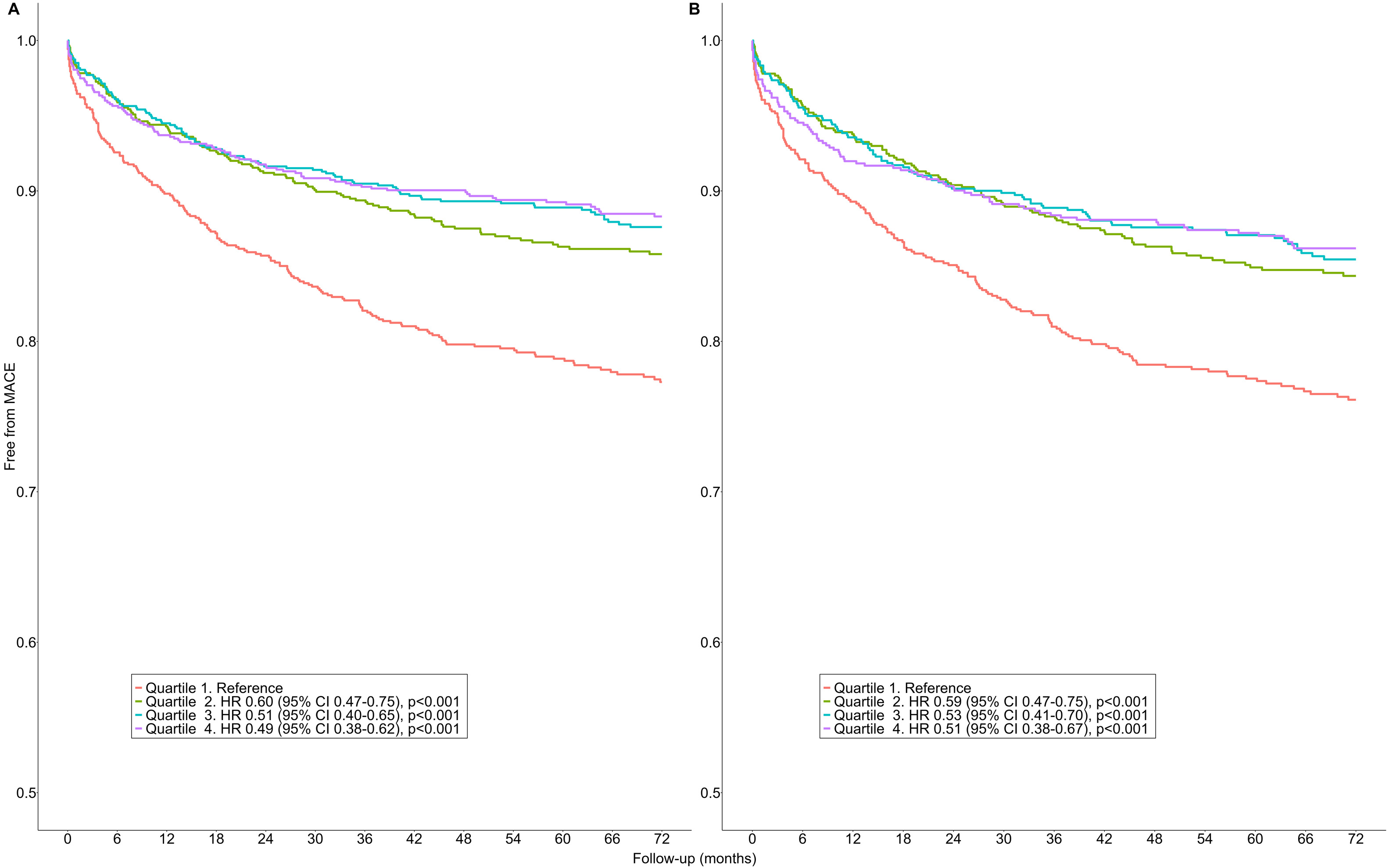

Supplement: Supplementary file 5 [file mmc5.jpg]
