# Supplementary material for: Impact of patient phenotype on the relationship between accelerometer-derived physical activity and cardiovascular events in atrial fibrillation
Source: Am J Prev Cardiol. 2025 Dec 5;25:101362. doi: 10.1016/j.ajpc.2025.101362 (PMC12743516; doi:10.1016/j.ajpc.2025.101362)
Supplement: Supplementary file 6 [file mmc6.docx]

| **Table S1.** Characteristics of the individuals with atrial fibrillation according to their clinical phenotypes | | | | |
| --- | --- | --- | --- | --- |
|  | Overall  (n=4,858) | ‘low risk’ Cluster 1  (n=2,275) | ‘high risk’ Cluster 2  (n=2,583) | P-values |
| **Characteristics** | | | | |
| MACE, n (%) | 764 (16) | 156 (7) | 608 (24) | **<0.001** |
| Age, years | 63 [58, 66] | 60 [55, 65] | 64 [61, 67] | **<0.001** |
| Female sex, n (%) | 1788 (37) | 776 (34) | 1012 (39) | **<0.001** |
| White background, n (%) | 4768 (98) | 2236 (98) | 2532 (98) | **<0.001** |
| BMI, kg/m^2^ | 27.2 [24.6, 30.5] | 26.9 [24.3, 30] | 27.6 [24.9, 31] | **<0.001** |
| DBP, mmHg | 82 [75, 89] | 82 [75, 90] | 82 [75, 89] | 0.05 |
| SBP, mmHg | 142 [130, 156] | 140 [128, 154] | 144 [131, 158] | **<0.001** |
| eGFR, mL/min/1.73m^2^ | 98.7 [93.4, 103.1] | 100.7 [95.6, 105.8] | 97.6 [91.3, 100.7] | **0.003** |
| Cystatin C, mg/L | 0.9 [0.8, 1] | 0.9 [0.8, 1] | 1 [0.9, 1.1] | **<0.001** |
| Haematocrit, % | 41.9 [39.6, 44.2] | 42.2 [39.9, 44.3] | 41.8 [39.4, 44] | **<0.001** |
| Gamma-glutamyl transferase, U/L | 29.2 [20.6, 46.7] | 29.6 [20.3, 48.6] | 28.9 [20.8, 43.8] | **0.03** |
| HDL, mmol/L | 1.2 [1, 1.4] | 1.3 [1.1, 1.5] | 1.2 [1, 1.4] | **<0.001** |
| LDL, mmol/L | 1.6 [1.3, 1.9] | 1.6 [1.4, 1.9] | 1.6 [1.3, 1.9] | **0.02** |
| Alcohol - daily use, n (%) | 1172 (24) | 843 (37) | 329 (13) | **<0.001** |
| Tobacco  Current, n (%) | 281 (6) | 158 (7) | 123 (5) | **0.04** |
| Tobacco - Previous, n (%) | 2195 (45) | 970 (43) | 1225 (47) | **<0.001** |
| Tobacco - Never, n (%) | 2365 (49) | 1140 (50) | 1225 (47) | 0.08 |
| **Medication** | | | | |
| Anti-hypertensive medication use, n (%) | 1567 (32) | 573 (25) | 994 (38) | **<0.001** |
| Cholesterol lowering medication use, n (%) | 1396 (29) | 528 (23) | 868 (34) | **<0.001** |
| Insulin use, n (%) | 51 (1) | 11 (0.5) | 40 (2) | **<0.001** |
| OAC use, n (%) | 441 (9) | 188 (8) | 253 (10) | **0.002** |
| **Cardiovascular risk factors** | | | | |
| Heart failure, n (%) | 355 (0.2) | 53 (2) | 302 (12) | **<0.001** |
| Type 2 diabetes, n (%) | 412 (8) | 91 (4) | 321 (12) | **<0.001** |
| Hypertension, n (%) | 7 (0.1) | 2 (0.09) | 5 (0.2) | 0.26 |
| **Comorbidities** | | | | |
| History of ischemic heart disease, n (%) | 1428 (29) | 276 (12) | 1152 (45) | **<0.001** |
| History of peripheral artery disease, n (%) | 178 (4) | 3 (0.1) | 175 (7) | **<0.001** |
| History of hypothyroidism, n (%) | 447 (9) | 85 (4) | 362 (14) | **<0.001** |
| History of hyperthyroidism, n (%) | 105 (2) | 39 (2) | 66 (3) | **0.008** |
| GI bleeding history, n (%) | 268 (6) | 93 (4) | 175 (7) | **<0.001** |
| History of stroke, n (%) | 657 (14) | 88 (4) | 569 (22) | **<0.001** |
| Intraventricular conduction: LBBB, n (%) | 200 (4%) | 46 (2) | 154 (6) | **<0.001** |
| Electrocardiographic evidence of LVH, n (%) | 193 (4%) | 40 (2) | 153 (6) | **<0.001** |
| Dementia, n (%) | 79 (2%) | 14 (0.6) | 65 (3) | **<0.001** |
| History of CABG, n (%) | 269 (6%) | 13 (0.6) | 256 (10) | **<0.001** |
| History of PCI, n (%) | 92 (2%) | 7 (0.3) | 85 (3) | **<0.001** |
| Cardioversion, n (%) | 901 (19%) | 542 (24) | 359 (14) | **<0.001** |
| Catheter ablation, n (%) | 708 (15%) | 466 (20) | 242 (9) | **<0.001** |
| Pacemaker, n (%) | 22 (0.5%) | 1 (0.04) | 21 (0.8) | **<0.001** |
| Renal replacement therapy/kidney transplantation, n (%) | 17 (0.3%) | 0 (0) | 17 (0.7) | **<0.001** |
| Anaemia, n (%) | 4858 (100%) | 2275 (100) | 2583 (100) | **<0.001** |
| Malignancy, n (%) | 532 (23%) | 532 (23) | 490 (19) | 0.19 |
| Sleep apnoea, n (%) | 218 (4%) | 110 (5) | 108 (4) | 0.89 |
| COPD, n (%) | 380 (8%) | 65 (3) | 315 (12) | **<0.001** |
| CKD, n (%) | 553 (11%) | 77 (3) | 476 (18) | **<0.001** |
| **Physical activity** | | | | |
| MVPA, min/week | 98 [69, 134] | 107 [76, 143] | 91 [62, 127] | **<0.001** |
| LPA, min/week | 291 [250, 336] | 291 [253, 336] | 291 [248, 337] | **0.04** |
| Sedentary, min/week | 1091 [1025, 1164] | 1082 [1018, 1152] | 1102 [1030, 1174] | **0.03** |
| Continuous variables are expressed as median [IQR]; dichotomous variables are expressed as number and percentage. MACE : major adverse cardiovascular events; BMI: body mass index; DBP: diastolic blood pressure; SBP: systolic blood pressure; eGFR: estimated glomerular filtration rate; HDL: high-density lipoprotein; LDL: low-density lipoprotein; OAC: Oral anticoagulant; GI bleeding: gastro-intestinal bleeding; LBBB: left Bundle branch block; LVH: left ventricular hypertrophy; CABG: coronary artery bypass graft; PCI: percutaneous coronary intervention; COPD: chronic obstructive pulmonary disease; CKD: chronic kidney disease; MVPA: moderate-vigorous physical activity; LPA: light physical activity. | | | | |

| **Table S2.** Characteristics of the individuals with atrial fibrillation without rhythm control procedures according to their physical activity volume | | | | | | |  |
| --- | --- | --- | --- | --- | --- | --- | --- |
|  | Overall  (n=3,504) | Quartile 1  (n=876) | Quartile 2  (n=876) | Quartile 3  (n=876) | Quartile 4  (n=876) | P-values | |
| **Characteristics** | | | | | | |  |
| MACE, n (%) | 526 (15) | 195 (22)^a,b,c^ | 122 (14) | 108 (12) | 101 (12) | **<0.001** | |
| Age, years | 62 [59, 66] | 63 [61, 67]^a,b,c^ | 62 [60, 67]^b,c^ | 62 [59, 66]^c^ | 60 [56, 65] | **<0.001** | |
| Female sex, n (%) | 1398 (40) | 341 (39)^c^ | 327 (37)^c^ | 352 (40) | 378 (43) | 0.08 | |
| White background, n (%) | 3440 (98) | 866 (99) | 857 (98) | 860 (98) | 857 (98) | >0.99 | |
| BMI, kg/m^2^ | 28.1 [24.6, 30.6] | 30 [25.8, 33.2]^a,b,c^ | 28.5 [25.1, 31]^b,c^ | 27.4 [24.3, 29.8]^c^ | 26.4 [23.6, 28.8] | **<0.001** | |
| DBP, mmHg | 82 [75, 89] | 83 [75, 91]^c^ | 83 [76, 89]^c^ | 82 [75, 88] | 81 [74, 89] | **0.03** | |
| SBP, mmHg | 144 [130, 157] | 146 [131, 160]^b,c^ | 145 [132, 156]^c^ | 144 [130, 157]^c^ | 141 [127, 153] | **<0.001** | |
| eGFR, mL/min/1.73m^2^ | 96.3 [93, 102.9] | 93.2 [88.5, 100.4]^a,b,c^ | 96.1 [93.4, 101.7]^c^ | 97.2 [94.1, 102.9] | 98.9 [95.4, 105.6] | **0.01** | |
| Cystatin C, mg/L | 1 [0.8, 1] | 1 [0.9, 1.1]^a,b,c^ | 1 [0.9, 1]^b,c^ | 0.9 [0.8, 1]^c^ | 0.9 [0.8, 1] | **<0.001** | |
| Haematocrit, % | 41.7 [39.4, 44] | 41.7 [39.4, 44] | 42 [39.8, 44.3]^c^ | 41.7 [39.3, 43.9] | 41.4 [39.1, 43.6] | **0.02** | |
| Gamma-glutamyl transferase, U/L | 41.3 [20.3, 46] | 44.8 [22.1, 49.6]^b,c^ | 44.6 [20.6, 47.8]^b,c^ | 39.8 [20.4, 46] | 36.1 [18.9, 41.2] | **<0.001** | |
| HDL, mmol/L | 1.3 [1, 1.5] | 1.3 [1, 1.4]^c^ | 1.2 [1, 1.4]^c^ | 1.3 [1, 1.5] | 1.3 [1.1, 1.5] | 0.05 | |
| LDL, mmol/L | 1.6 [1.3, 1.9] | 1.6 [1.3, 1.9]^b,c^ | 1.6 [1.3, 1.9] | 1.7 [1.4, 2] | 1.7 [1.4, 1.9] | 0.08 | |
| Alcohol - daily use, n (%) | 813 (23) | 204 (23) | 191 (22) | 226 (26) | 192 (22) | 0.27 | |
| Tobacco  Current, n (%) | 199 (6) | 69 (8)^b,c^ | 57 (7)^b,c^ | 37 (4) | 36 (4) | **0.001** | |
| Previous, n (%) | 1586 (45) | 434 (50)^a,c^ | 380 (43) | 398 (45) | 374 (43) | 0.14 | |
| Never, n (%) | 1704 (49) | 370 (42)^a,b,c^ | 434 (50) | 440 (50) | 460 (53) | **0.01** | |
| **Medication** | | | | | | |  |
| Anti-hypertensive medication use, n (%) | 1154 (33) | 385 (44)^a,b,c^ | 305 (35)^b,c^ | 277 (32)^c^ | 187 (21) | **<0.001** | |
| Cholesterol lowering medication use, n (%) | 1044 (30) | 331 (38)^a,b,c^ | 283 (32)^b,c^ | 242 (28)^c^ | 188 (21) | **<0.001** | |
| Insulin use, n (%) | 41 (1) | 15 (2)^c^ | 13 (1) | 8 (0.9) | 5 (0.6) | 0.11 | |
| Warfarin use, n (%) | 240 (7) | 70 (8) | 61 (7) | 54 (6) | 55 (6) | 0.44 | |
| **Cardiovascular risk factors** | | | | | | |  |
| Heart failure, n (%) | 251 (7) | 106 (12)^a,b,c^ | 55 (6) | 47 (5) | 43 (5) | **<0.001** | |
| Type 2 diabetes, n (%) | 323 (9) | 157 (18)^a,b,c^ | 87 (10)^b,c^ | 54 (6)^c^ | 25 (3) | **<0.001** | |
| Hypertension, n (%) | 3 (0.09) | 0 (0) | 1 (0.1) | 2 (0.2) | 0 (0) | 0.30 | |
| **Comorbidities** | | | | | | |  |
| History of ischemic heart disease, n (%) | 1021 (29) | 346 (39)^a,b,c^ | 267 (30)^b,c^ | 222 (25)^c^ | 186 (21) | **<0.001** | |
| History of peripheral artery disease, n (%) | 132 (4) | 64 (7)^a,b,c^ | 32 (4) | 18 (2) | 18 (2) | **<0.001** | |
| History of hypothyroidism, n (%) | 349 (10) | 115 (13)^b,c^ | 89 (10) | 75 (9) | 70 (8) | **0.003** | |
| History of hyperthyroidism, n (%) | 72 (2) | 27 (3)^c^ | 20 (2)^c^ | 17 (2) | 8 (0.9) | **0.02** | |
| GI bleeding history, n (%) | 199 (6) | 64 (7)^b,c^ | 57 (7) | 40 (5) | 38 (4) | **0.02** | |
| History of stroke, n (%) | 491 (14) | 199 (23)^a,b,c^ | 103 (12)^c^ | 113 (13)^c^ | 76 (9) | **<0.001** | |
| Intraventricular conduction: LBBB, n (%) | 140 (4%) | 50 (6)^c^ | 35 (4) | 35 (4) | 20 (2) | **0.005** | |
| Electrocardiographic evidence of LVH, n (%) | 104 (3%) | 36 (4)^b^ | 21 (2) | 17 (2) | 30 (3) | **0.04** | |
| Dementia, n (%) | 65 (2%) | 29 (3)^a,b,c^ | 13 (1) | 12 (1) | 11 (1) | **0.004** | |
| History of CABG, n (%) | 220 (6%) | 72 (8)^b,c^ | 63 (7)^c^ | 49 (6) | 36 (4) | **0.003** | |
| History of PCI, n (%) | 77 (2%) | 31 (4)^a,c^ | 14 (2) | 20 (2) | 12 (1) | **0.01** | |
| Pacemaker, n (%) | 17 (0.5%) | 2 (0.2)^b^ | 2 (0.2)^b^ | 10 (1) | 3 (0.3) | **0.01** | |
| Renal replacement therapy/kidney transplantation, n (%) | 15 (0.4%) | 8 (0.9)^b^ | 3 (0.3) | 1 (0.1) | 3 (0.3) | 0.07 | |
| Anaemia, n (%) | 3504 (100%) | 876 (100) | 876 (100) | 876 (100) | 876 (100) | >0.99 | |
| Malignancy, n (%) | 196 (22%) | 196 (22) | 203 (23) | 197 (22) | 169 (19) | 0.308 | |
| Sleep apnoea, n (%) | 149 (4%) | 65 (7)^a,b,c^ | 38 (4)^b^ | 18 (2) | 28 (3) | **<0.001** | |
| COPD, n (%) | 291 (8%) | 121 (14)^a,b,c^ | 66 (8)^c^ | 60 (7) | 44 (5) | **<0.001** | |
| CKD, n (%) | 430 (12%) | 196 (22)^a,b,c^ | 93 (11)^c^ | 79 (9) | 62 (7) | **<0.001** | |
| **Physical activity** | | | | | | |  |
| MVPA, min/week | 96 [68, 132] | 49 [36, 59]^a,b,c^ | 82 [75, 89]^b,c^ | 112 [104, 122]^c^ | 162 [144, 191] | **<0.001** | |
| LPA, min/week | 293 [251, 340] | 257 [215, 297]^a,b,c^ | 287 [248, 330]^b,c^ | 301 [268, 345]^c^ | 324 [287, 375] | **<0.001** | |
| Sedentary, min/week | 1093 [1025, 1167] | 1168 [1119, 1223]^a,b,c^ | 1105 [1060, 1152] ^b,c^ | 1060 [1017, 1111]^c^ | 1009 [955, 1100] | **<0.001** | |
| Continuous variables are expressed as median [IQR]; dichotomous variables are expressed as number and percentage.  MACE : major adverse cardiovascular events; BMI: body mass index; DBP: diastolic blood pressure; SBP: systolic blood pressure; eGFR: estimated glomerular filtration rate; HDL: high-density lipoprotein; LDL: low-density lipoprotein; GI bleeding: gastro-intestinal bleeding; LBBB: left Bundle branch block; LVH: left ventricular hypertrophy; CABG: coronary artery bypass graft; PCI: percutaneous coronary intervention; COPD: chronic obstructive pulmonary disease; CKD: chronic kidney disease; MVPA: moderate-vigorous physical activity; LPA: light physical activity.  ^a^: vs Quartile 2, p<0.05; ^b^: vs Quartile 3, p<0.05 ; ^c^: vs Quartile 4, p<0.05. | | | | | | |  |

| **Table S3.** Clinical factor definitions | | | | |
| --- | --- | --- | --- | --- |
| **Outcomes** | **Data fields** | **Field Name** | **Data codes** | **Data code definitions** |
| Atrial fibrillation/  Flutter | 41202 | Diagnoses – Main ICD10 | I48, I48.0, I48.1, I48.2,  I48.3, I48.4, I48.9 | Atrial fibrillation and flutter; paroxysmal atrial fibrillation; persistent atrial fibrillation; chronic atrial fibrillation; typical atrial flutter; atypical atrial flutter; atrial fibrillation and atrial flutter, unspecified |
| Rhythm control procedures | 41200 | Operative Procedures – Main OPCS4 | K57.1, K57.2, K62.1, K62.2, K62.3, K62.4, K62.5, K52.1, K57.1, K57.2, K57.4, K57.5, X50.1 | Percutaneous transluminal ablation of atrioventricular node, Percutaneous transluminal ablation of conducting system of heart NEC, Percutaneous transluminal ablation of pulmonary vein to left atrium conducting system, Percutaneous transluminal ablation of atrial wall for atrial flutter, Percutaneous transluminal ablation of conducting system of heart for atrial flutter NEC, Percutaneous transluminal internal cardioversion NEC, Open ablation of atrioventricular node, Percutaneous transluminal ablation of atrioventricular node, Percutaneous transluminal ablation of conducting system of heart NEC, Percutaneous transluminal ablation of accessory pathway, Percutaneous transluminal ablation of atrial wall NEC, Direct current cardioversion |
| Major Adverse Cardiovascular Events | 41202 | Diagnoses – Main ICD10 | I60, I61, I62, I63, I64, G45, I50, I21, I22, I25, I46, I73, I74, 40000 | Haemorrhage stroke, ischaemic stroke, heart failure, acute myocardial infarction, myocardial infarction, ischaemic heart disease, cardiac arrest |
| Cardiovascular risk factors | 41202 | Diagnoses – Main ICD10 | E11, I10, I50 | Type 2 diabetes, hypertension, heart failure |
| Smoking status | 20116 | Smoking status |  |  |
| Alcohol status | 1558 | Alcohol intake frequency |  |  |
| Age | 21022 | Age at recruitment |  |  |
| Ethnicity | 21000 | Ethnic |  |  |
| Body mass index | 23104 | Body mass index |  |  |
| Medication | 6177 | Medication for cholesterol, blood pressure or diabetes |  | Cholesterol lowering medication, blood pressure medication, insulin, warfarin use |

| **STROBE Statement** – checklist of items that should be included in reports of observational studies | | | | |
| --- | --- | --- | --- | --- |
|  | **Item No** | **Recommendation** | **Page No** |  |
| **Title and abstract** | 1 | (*a*) Indicate the study’s design with a commonly used term in the title or the abstract | 1 |  |
|  |  | (*b*) Provide in the abstract an informative and balanced summary of what was done and what was found | 1 |  |
| Introduction |  |  |  |  |
| Background/rationale | 2 | Explain the scientific background and rationale for the investigation being reported | 2,3 |  |
| Objectives | 3 | State specific objectives, including any prespecified hypotheses | 3 |  |
| Methods | | | | |
| Study design | 4 | Present key elements of study design early in the paper | 3,4 |  |
| Setting | 5 | Describe the setting, locations, and relevant dates, including periods of recruitment, exposure, follow-up, and data collection | 3-6 |  |
| Participants | 6 | (*a*) *Cohort study*—Give the eligibility criteria, and the sources and methods of selection of participants. Describe methods of follow-up  *Case-control study*—Give the eligibility criteria, and the sources and methods of case ascertainment and control selection. Give the rationale for the choice of cases and controls  *Cross-sectional study*—Give the eligibility criteria, and the sources and methods of selection of participants | 3,4 |  |
|  |  | (*b*) *Cohort study*—For matched studies, give matching criteria and number of exposed and unexposed  *Case-control study*—For matched studies, give matching criteria and the number of controls per case | NA |  |
| Variables | 7 | Clearly define all outcomes, exposures, predictors, potential confounders, and effect modifiers. Give diagnostic criteria, if applicable | 5,6 |  |
| Data sources/ measurement | 8* | For each variable of interest, give sources of data and details of methods of assessment (measurement). Describe comparability of assessment methods if there is more than one group | 5,6 |  |
| Bias | 9 | Describe any efforts to address potential sources of bias | 7 |  |
| Study size | 10 | Explain how the study size was arrived at | Supplemental Figure 1 |  |
| Quantitative variables | 11 | Explain how quantitative variables were handled in the analyses. If applicable, describe which groupings were chosen and why | 4,5 |  |
| Statistical methods | 12 | (*a*) Describe all statistical methods, including those used to control for confounding | 6,7 |  |
|  |  | (*b*) Describe any methods used to examine subgroups and interactions | 6,7 |  |
|  |  | (*c*) Explain how missing data were addressed | NA |  |
|  |  | (*d*) *Cohort study*—If applicable, explain how loss to follow-up was addressed  *Case-control study*—If applicable, explain how matching of cases and controls was addressed  *Cross-sectional study*—If applicable, describe analytical methods taking account of sampling strategy | NA |  |
|  |  | (*e*) Describe any sensitivity analyses | Figure S4 and Table S2 |  |
| **Results** | | | | |
| Participants | 13* | (a) Report numbers of individuals at each stage of study—eg numbers potentially eligible, examined for eligibility, confirmed eligible, included in the study, completing follow-up, and analysed | 8 |  |
|  |  | (b) Give reasons for non-participation at each stage | 8 |  |
|  |  | (c) Consider use of a flow diagram | Supplemental Figure 1 |  |
| Descriptive data | 14* | (a) Give characteristics of study participants (eg demographic, clinical, social) and information on exposures and potential confounders | Tables 1 and 2 |  |
|  |  | (b) Indicate number of participants with missing data for each variable of interest | Supplemental Figure 1 |  |
|  |  | (c) *Cohort study*—Summarise follow-up time (eg, average and total amount) | 8 |  |
| Outcome data | 15* | *Cohort study*—Report numbers of outcome events or summary measures over time | 8-10, Figures 1-2 |  |
|  |  | *Case-control study—*Report numbers in each exposure category, or summary measures of exposure | *NA* |  |
|  |  | *Cross-sectional study—*Report numbers of outcome events or summary measures | *NA* |  |
| Main results | 16 | (*a*) Give unadjusted estimates and, if applicable, confounder-adjusted estimates and their precision (eg, 95% confidence interval). Make clear which confounders were adjusted for and why they were included | 8-10, Figures 1-2 |  |
|  |  | (*b*) Report category boundaries when continuous variables were categorized | NA |  |
|  |  | (*c*) If relevant, consider translating estimates of relative risk into absolute risk for a meaningful time period | NA |  |
| Other analyses | 17 | Report other analyses done—eg analyses of subgroups and interactions, and sensitivity analyses | Figure 3, and supplemental |  |
| **Discussion** | | | | |
| Key results | 18 | Summarise key results with reference to study objectives | 12 |  |
| Limitations | 19 | Discuss limitations of the study, taking into account sources of potential bias or imprecision. Discuss both direction and magnitude of any potential bias | 14-15 |  |
| Interpretation | 20 | Give a cautious overall interpretation of results considering objectives, limitations, multiplicity of analyses, results from similar studies, and other relevant evidence | 12-14 |  |
| Generalisability | 21 | Discuss the generalisability (external validity) of the study results | 12-13 |  |
| **Other information** | | | | |
| Funding | 22 | Give the source of funding and the role of the funders for the present study and, if applicable, for the original study on which the present article is based | 16 |  |

**Figures’ legends**

**Figure S1.** Flow diagram of the cohorts included in the primary (A), sub-group, and sensitivity analyses (B)

**Figure S2.** Dendogram hierarchical clustering for individuals with atrial fibrillation cluster.

**Figure S3.** Survival analyses from major adverse cardiovascular events risks in individuals with atrial fibrillation according to their clinical phenotypes’ stratification.

MACE: major adverse cardiovascular events.

**Figure S4.** Survival analyses from major adverse cardiovascular events risks in individuals with atrial fibrillation without rhythm control procedures according to physical activity volume.

Unadjusted (A) and adjusted (B) for age, sex, ethnicity sedentarity, body mass index, alcohol and tobacco use, and cardiovascular risk factors for atrial fibrillation.

MACE: major adverse cardiovascular events.

P-values refer to the comparison to the reference group (*i.e*., ‘Quartile 1’)
